# Tubulointerstitial nephritis and uveitis syndrome complicated by IgA nephropathy and Graves’ disease: a case report

**DOI:** 10.1186/1752-1947-8-305

**Published:** 2014-09-12

**Authors:** Yoshinosuke Shimamura, Takahiro Tsushima, Norihito Moniwa, Koichi Hasegawa, Yayoi Ogawa, Hideki Takizawa

**Affiliations:** 1 Department of Nephrology, Teine Keijinkai Medical Center, 1-12, Maeda, Teine-ku, Sapporo 006-8555, Hokkaido, Japan; 2 Hokkaido Renal Pathology Center, Sapporo IT Front Building, 196, kita9 nishi15, 20, Chuo-ku, Sapporo 060-0000, Hokkaido, Japan

**Keywords:** Corticosteroids, Graves’ disease, IgA nephropathy, TINU syndrome

## Abstract

**Introduction:**

Tubulointerstitial nephritis and uveitis syndrome is a disorder characterized by a combination of acute tubulointerstitial nephritis and uveitis. Immunoglobulin A nephropathy is defined by the presence of immunoglobulin A deposits in glomerular mesangial areas. In this report, we describe a rare case of tubulointerstitial nephritis and uveitis syndrome complicated by immunoglobulin A nephropathy and Graves’ disease, which was successfully treated with corticosteroids. To the best of our knowledge, this is the first time such a case has been documented since tubulointerstitial nephritis and uveitis syndrome was first described.

**Case presentation:**

A 64-year-old Japanese woman presented with tubulointerstitial nephritis and uveitis syndrome accompanied by immunoglobulin A nephropathy and Graves’ disease. She had renal dysfunction, proteinuria, and hematuria. Two weeks after her admission, she developed anterior chamber uveitis. She received corticosteroids, resulting in significant clinical improvement.

**Conclusion:**

Tubulointerstitial nephritis and uveitis syndrome is a relatively uncommon cause of tubulointerstitial nephritis. Clinicians should recognize that tubulointerstitial nephritis and uveitis syndrome with immunoglobulin A nephropathy can occur in the presence of Graves’ disease. Additionally, this report may provide important clues in terms of the management of a concomitant case of these diseases.

## Introduction

Tubulointerstitial nephritis and uveitis (TINU) syndrome is defined by acute tubulointerstitial nephritis associated with uveitis that occurs either simultaneously, prior to, or following the onset of renal dysfunction [1]. By contrast, immunoglobulin A (IgA) nephropathy is the most prevalent primary chronic glomerulonephritis in Japan as well as worldwide [2], and is characterized by IgA and C3 deposition in glomerular mesangial regions. According to the Japan Renal Biopsy Registry, there were 239 cases of IgA nephropathy in 2007 and 424 cases in 2008 [3]. The clinical course of IgA nephropathy is variable, but one quarter of patients will have end-stage renal diseases after 20 years, and a further 20% will have progressive impairment of renal function [4]. In both TINU and IgA nephropathy, the pathogenesis is thought to be an autoimmune process that might involve humoral and cellular autoimmunity [5,6]. The standard treatment of IgA nephropathy is corticosteroids, resulting in preserving renal function over the long term [7]. The standard therapy of TINU syndrome is still not clear.

We describe the case of a patient with TINU syndrome complicated by IgA nephropathy and Graves’ disease, which was treated successfully with corticosteroids, leading to a decline in proteinuria and normalized renal function.

## Case presentation

A 64-year-old Japanese woman presented with a sore throat and hematuria of three days duration. She reported fever, chills, and fatigue. All other review of systems showed no other abnormalities. Besides Graves’ disease, she had hypertension and was taking propylthiouracil, amlodipine, and candesartan; she had no known allergies. She had never smoked or used recreational drugs and only occasionally drank alcohol. She had no family history of kidney disease. A physical examination revealed the following vital signs: temperature, 37.1°C; heart rate, 94 beats per minute; blood pressure, 115/80mmHg; respiratory rate, 12 breaths per minute; and O2 saturation, 98% on room air. A head and neck examination showed her oropharynx to be erythematous with swollen tonsils and pus. She had multiple, tender, mobile, 5mm bilateral cervical lymphadenopathy. The patient denied short of breath, sputum production, difficulty swallowing. Her thyroid, chest, and abdominal examinations showed no abnormalities, along with the rest of her examination.

One year prior to her presentation, our patient’s renal function and urine analysis were as follows: blood urea nitrogen (BUN), 12.1mg/dL; creatinine (Cr), 1.1mg/dL; and estimated glomerular filtration rate (eGFR), 59mL/min per 1.73m^2^ according to the Modification of Diet in Renal Disease equation. Her urinary sediment at that time revealed 10 to 19 erythrocytes and one to four leukocytes per high-power field, and no casts were noted. On her current admission, significant laboratory findings included a white blood cell count of 23,310 cells/μL, BUN of 15.4mg/dL, Cr of 1.4mg/dL, and eGFR of 51mL/min per 1.73m^2^ according to the Modification of Diet in Renal Disease equation. Findings from a thyroid function panel were within normal limits. Her erythrocyte sedimentation rate (ESR) was 62mm/h. A rapid strep test on a throat culture was positive for *Streptococcus pyogenes*, for which she was given ceftriaxone. Urine analysis on a spot urine analysis revealed a protein/creatinine ratio of 2.0g/g.Cr. Her urinary protein was 2.6g/day; her urinary sediment revealed 50 to 99 erythrocytes and one to four leukocytes per high-power field, and no casts were noted.

**Figure 1:**
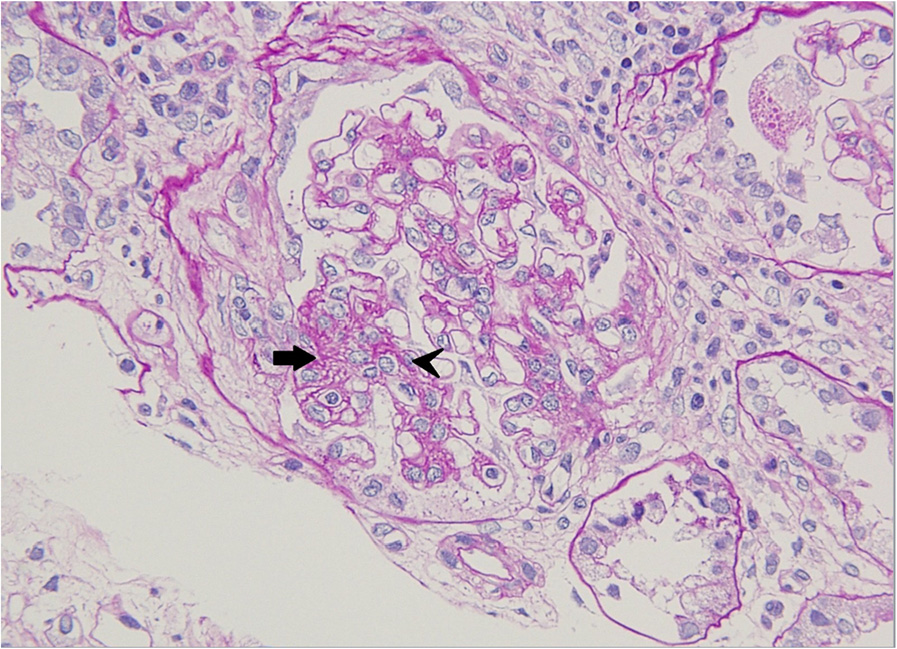
**Renal biopsy (periodic acid silver methenamine-Masson trichrome, ×40).** Mild proliferation of mesangial cells (arrow head), with three or more cells per mesangial area, and increase of mesangial matrix (arrow).

**Figure 2:**
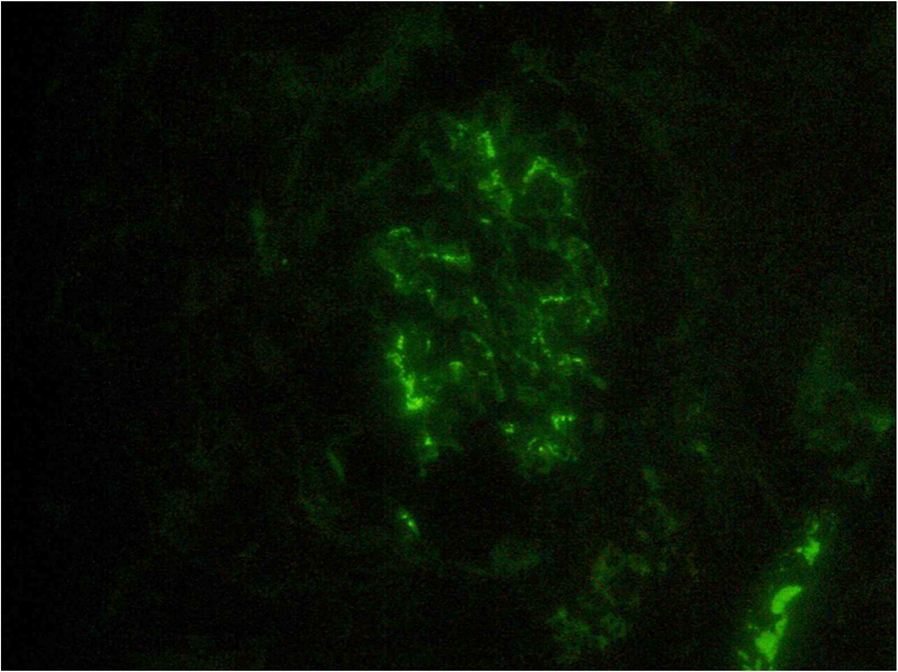
**Renal biopsy (immunofluorescence, ×400).** Diffuse mesangial deposit of immunoglobulin A.

**Figure 3:**
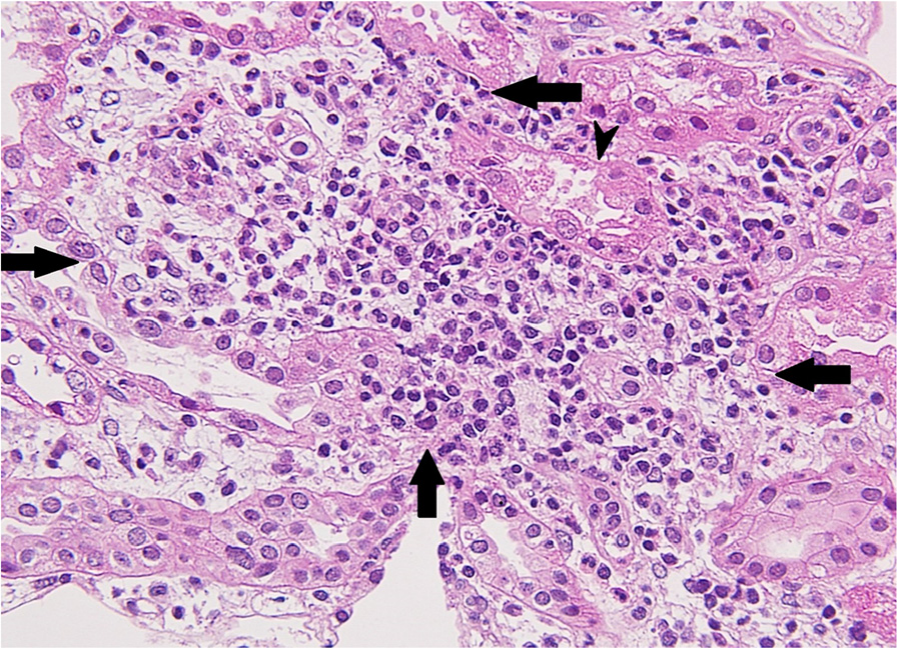
**Renal biopsy (hematoxylin and eosin, ×40).** In the renal tubular interstitium, marked immune cell infiltration, composed of mainly lymphocytes and edema, was observed (arrow). In addition, tubular atrophy was observed (arrow head).

Two weeks later, our patient developed bilateral ciliary injection and photophobia; slit lamp examination revealed anterior chamber uveitis. Laboratory findings were as follows: BUN, 26mg/dL; Cr, 2mg/dL; anti-nuclear antibody titer, 1:320; and IgA, 497mg/dL (normal range: 90 to 400mg/dL). A urine analysis at this time revealed protein of 3.2g/day and β2-microglobulin of 1347/μL (normal 0-250/μL). Histological examination of a kidney biopsy specimen showed one cellular crescent, increased mesangial cell proliferation (Figure 1), and deposition of IgA (Figure 2) in the glomeruli, consistent with IgA nephropathy. The interstitium, however, showed tubular atrophy, edematous changes, and infiltration of granular leukocytes (Figure 3). Along with the uveitis, her elevated ESR, the excretion of urinary β2-microglobulin, and the histology of the kidney specimen were highly suggestive of TINU syndrome. Given her diagnosis of TINU syndrome [1] complicated by IgA nephropathy, she was given methylprednisolone for three days with subsequent oral prednisolone for two months, with clinical improvement.

## Discussion

In this case, we made two important clinical observations. First, this is the first reported case of IgA nephropathy complicated by TINU syndrome with underlying Graves’ syndrome. IgA nephropathy is often associated with other conditions, particularly with hepatic diseases and autoimmune diseases [8]. Ku *et al*. reported the case of a patient with Graves’ disease complicated by IgA nephropathy but not TINU syndrome [9]. Several researchers have reported the concurrence of TINU syndrome and IgA nephropathy with [10,11] or without underlying autoimmune diseases [12-14]. Therefore, we need to hold a high clinical suspicion for other autoimmune-mediated diseases in patients with IgA nephropathy. However, to the best of our knowledge, there are no previously reported cases of IgA nephropathy complicated by TINU syndrome with underlying Graves’ disease.

Our second observation is that corticosteroid therapy significantly improved both our patient’s IgA nephropathy and TINU syndrome. It appears beneficial in moderate or severe cases of IgA nephropathy [15] whereas it is still not clear that steroid therapy is required for the recovery of tubulointerstitial nephritis. In terms of IgA nephropathy, Kidney Disease Improving Global Outcomes guidelines recommend six months of glucocorticoids when there is persistent proteinuria is over 1g/day, and GFR greater than 50mL/min per 1.73m^2^[15]. In regards to TINU, whether corticosteroids are beneficial for nephritis is still unknown. Some patients receive oral steroids to treat the renal dysfunction and the response is favorable [16,17], but other patients were reported to recover spontaneously without any drugs, including steroids [18,19]. In this case, we successfully treated both our patient’s renal function and uveitis.

This case report has limitations that must be noted. First, tubulointerstitial lesions can be associated with aggravation of IgA nephropathy. Second, the role of corticosteroids in the treatment of TINU syndrome remains to be defined. In the absence of prospective randomized double-blind trials, we are compelled to use evidence from small, uncontrolled series. However, we used corticosteroids in our patient because her renal function deteriorated.

We may speculate on the pathogenesis of this case as follows. Because the contribution of *Streptococcus* infection to the pathogenesis of IgA nephropathy has been reported [20], we can infer first of all that a *Streptococcus* infection triggered her humoral immune responses, causing IgA nephropathy. Subsequently, these reactions may have activated abnormal immune responses, with cellular and humoral interactions resulting in TINU syndrome.

## Conclusion

We report on a rare case of TINU complicated by IgA nephropathy and Graves’ disease, successfully treated with corticosteroids. Clinicians should be aware that TINU syndrome may complicate other autoimmune diseases (for example (11,12]). In addition, a multicenter prospective trial investigating the role of early steroid therapy in TINU is necessary.

## Consent

Written informed consent was obtained from the patient for publication of this case report and accompanying images. A copy of the written consent is available for review by the Editor-in-Chief of this journal.

## Abbreviations

BUN: blood urea nitrogen; Cr: creatinine; eGFR: estimated glomerular filtration rate; ESR: erythrocyte sedimentation rate; IgA: immunoglobulin A; TINU: tubulointerstitial nephritis with uveitis.

## Competing interests

The authors declare that they have no competing interests.

## Authors’ contributions

HT was a major contributor in writing the manuscript. TT, NM and KH analyzed and interpreted the patient data regarding the renal disease. YO performed the histological examination of the kidney. YS was mainly writing the manuscript. All authors read and approved the final manuscript.
